# 

**Published:** 1874-08

**Authors:** 


### CONTENTS:

### ORIGINAL COMMUNICATIONS.

ARTICLE I.—Dentistry. By Prof. P. H. Austen, M. A., M. D., D. D. S ........ 145
ARTICLE II.—Taking Upper Impressions of the Mouth. By Dr. Geo. S. Fouke........ 153
ARTICLE VIII.—The South Carolina State Dental Association........ 182

### SELECTED ARTICLES.

ARTICLE III.—The Use and Comparative Merits of Bichloride of Methylene as an Anæsthetic. By Benj F. Dawson, M. D........ 157
ARTICLE IV. Principal Causes of Constitutional Derangement in First Dentition. By Dr. J. Hardman........ 165
ARTICLE V.—On the Mode of Action of Iodine and its Preparations.—By Prof. Lee........ 171
ARTICLE VI.—The Most Efficacious Form of Arsenic for Devitalizing Dental Pulps. By Thos. Burgh, D. D. S. ........ 176
ARTICLE VII.—The Styloid Muscles and Anæsthetics By G W. Copeland, M. D........ 180

### EDITORIAL, ETC.

A Mew Method of Extracting Temporary Teeth........ 183
Poisoning from Corrosive Sublimate Generated by Amalgam Fillings........ 184

### MONTHLY SUMMARY.

Solubility of Arsenious Acid in Alcohol........ 186

Nickeling........ 187

Large Calculus ........ 188

Iodoform as a Topical Remedy........ 189

Remedy for Tooth-ache........ 189

A French Charlatan........ 190

Use of Iodic Acid by Hypodermic Injection........ 190

The Prevention Cicatrices........ 190

Death from a Dose of Chloral and Bromide of Potassium........ 190

Bone and Skin Grafting........ 191

Trichinæ........ 191

Death from Lancing Gums........ 191

Lusus Naturæ........ 191

Methyl-bichloride........ 191

